# Exploration of subgroups and predictors of health-promoting lifestyle among older adults in the community: a latent profile analysis

**DOI:** 10.3389/fpubh.2026.1583243

**Published:** 2026-02-26

**Authors:** Jianyi Bao, Shasha Li, Yuwei Lu, Guojing Guo, Yuecong Wang, Shufang Liao, Yue Li, Yingxue Xi, Xiaofang Song, Xinyu Yang

**Affiliations:** 1 School of Medicine and Nursing, Huzhou University, Huzhou, Zhejiang, China; 2 Department of Nursing, Huzhou Central Hospital, Huzhou, Zhejiang, China

**Keywords:** community, older adults, health promotion, healthy lifestyle, latent profile analysis

## Abstract

**Purpose:**

The health-promoting lifestyle has an impact on the quality of life of older people. This study aimed to explore the subgroup characteristics and predictors of health-promoting lifestyles among community-dwelling older adults.

**Methods:**

This study involved 503 community-dwelling Chinese older adults. Latent profile analysis was employed to identify subgroups of community-dwelling older adults with health-promoting lifestyles, and predictors affecting each subgroup were analyzed using univariate analysis and multiple logistic regression analysis.

**Results:**

Health-promoting lifestyles among community-dwelling older adults were identified in three categories: low health-promoting lifestyle group (32.80%), moderate health-promoting lifestyle-low health responsibility group (44.33%), and high health-promoting lifestyle-low stress management group (22.86%). Gender, the number of chronic diseases, smartphone use, residential status, monthly income, participation in geriatric activities, household registration type, medical insurance, friend networks, and health risk perception were predictors of subgroup membership.

**Conclusion:**

This study classified health-promoting lifestyles among community-residing older adults and identified predictors for each, helping to develop tailored health intervention programs.

## 1 Introduction

Population aging has become an irreversible global trend of our time. According to data from the National Bureau of Statistics of China, by the end of 2023, China’s population aged 60 and above had reached 297 million, accounting for 21.1% of the total population; those aged 65 and above numbered 217 million, representing 15.4% of the total population (1). China’s population aging exhibits dual characteristics of rapid pace and profound depth. Aging not only exposes older adults to a series of health risks—including physical decline, unhealthy lifestyles, multiple coexisting conditions, and functional impairment—but also places sustained pressure on socioeconomic systems and healthcare service frameworks (2–4). To address these challenges, the World Health Organization (WHO) defines healthy aging in its *Report on Aging and Health* as the ability to develop and maintain health-related functioning in later life (5). Achieving healthy aging contributes to improving the quality of life for older adults and extending healthy life expectancy. There is an urgent need to identify feasible and cost-effective interventions. Notably, The Lancet emphasizes that healthy lifestyles are key to achieving healthy aging (6). One study suggests that integrating multiple healthy lifestyles is associated with slower decline rates in physical, mental, cognitive, and social functioning in older adults during aging (7). Research by Rizzuto et al. indicates that older adults who maintain healthy lifestyle behaviors have a life expectancy 5.4 years longer than those with unhealthy lifestyle behaviors (8), demonstrating the positive impact of adhering to healthy habits on extending the life expectancy of older adults. It is noteworthy that unhealthy lifestyles—such as smoking, lack of exercise, poor dietary habits, and sleep disorders—have become major contributing factors to morbidity, mortality, and preventable complications among older people (9). Therefore, it is crucial to focus on the current status and characteristics of health-promoting lifestyles among older adults.

Health-Promoting Lifestyle (HPL) is a spontaneous and active healthy lifestyle, centered on enabling individuals achieve self-worth or personal fulfillment while maintaining and enhancing their health status (10). This lifestyle possesses a multidimensional structure encompassing six dimensions: self-actualization, health responsibility, physical exercise, nutritional management, interpersonal support, and stress coping (10). These dimensions represent the goals individuals strive for to achieve a healthier state. HPL plays a crucial role among older adults, as it effectively reduces the incidence of depression (11), lowers the prevalence of chronic diseases (12), and improves quality of life (13). Notably, previous studies have pointed out that community-living older adults’ health-promoting lifestyles suffer from the problem of “four low” characteristics: weak sense of self-health responsibility (14), limited social participation (15), inadequate social support and stress coping ability (16), and low sense of self-actualized worth (17). Previous studies have judged the level of health-promoting lifestyles in older adults by analyzing the total scale score or the scores of each dimension (18–20). However, these approaches are deficient in their inability to reflect inter-individual heterogeneity in the level of health-promoting lifestyles, and they ignore the interactions between health-promoting lifestyles within different individual levels. Instead, exploring differences in the level of health-promoting lifestyles of older individuals is a key point for effective identification and development of targeted interventions.

Compared with traditional variable-centered approaches, the key advantage of Latent Profile Analysis (LPA) lies in capturing unobserved heterogeneity in behavioral patterns within populations. LPA is an “individual-centered” analysis method that identifies latent groups with similar characteristics through model fitting estimation based on the response probabilities on each dimension or item of the study variable, and uses latent class variables to explain the association between dominant continuous variables. The samples were divided into different categories to achieve local independence between the displayed variables (21–23). This person-centered approach can effectively identify the types and characteristics of HPL and facilitate the implementation of precise interventions for groups with the same characteristics, which is beneficial to save manpower, material resources, and investment in health promotion programs (24, 25).

LPA has been widely applied in gerontology. Existing studies have identified distinct group types with significant differences in single-dimensional health behaviors or psychosocial traits such as dietary patterns (26), 24-h movement behaviors (27), mental health literacy (28), and social participation through this method (29), providing an important perspective for understanding the heterogeneity the older population. However, these studies have mostly focused on single-dimensional indicators and failed to comprehensively capture the complex multi-dimensional concept of a health-promoting lifestyle. According to Pender’s Health Promotion Model theory (30), an individual’s adoption of a health-promoting lifestyle is a comprehensive result of the interaction of individual characteristics, cognitive and emotional factors, and behavioral outcomes. This implies that there may be multi-dimensional interactions within a health-promoting lifestyle, which is of significant theoretical importance for revealing the heterogeneity of health-promoting lifestyles among older adults. Although Guo et al. (31) used LPA technology to identify HPL in the hospitalized patient population at high risk of stroke. They were divided into three groups: the self-actualization deficiency group, the social anxiety group, and the health responsibility deficiency group. However, since the research subjects were specific clinical populations, its conclusions are difficult to generalize to the more general older population in the community. At present, there is no study that has evaluated the health-promoting lifestyle of older adults in Chinese communities using LPA, and there is still a gap in the understanding of the intrinsic heterogeneity of this group. This is not conducive to the in-depth implementation of health lifestyle promotion plans such as home-based senior care, community integration, mutual assistance for seniors, and smart aged care.

Previous research has shown that the factors that influence health-promoting lifestyles among older people are not homogeneous. Instead, they are often characterized by diversity. Sociodemographic characteristics such as gender (32, 33), age (34), marriage (35), monthly income (36), the number of diseases (32, 37), and participation in social activities (33) are predictors of health-promoting lifestyle in older adults. In addition, Wu et al. found that the lack of a network of friends was associated with low levels of healthy lifestyles among older adults (38). In addition, a study has shown that a higher level of perceived health risk is associated with a higher level of health-promoting behavior (39). However, the heterogeneity of health-promoting lifestyles among older adults means that there may be differences in the factors influencing different categories. Therefore, it is essential to explore the interactive effects of multiple factors on the health-promoting lifestyles of different categories of community-based older adults.

This study employed LPA to identify subtypes of HPL among community-dwelling older adults and to explore the influencing factors associated with different latent profiles of such lifestyles, aiming to provide a theoretical foundation for developing targeted intervention programs. Based on the theory of health promotion models and previous research, we hypothesized that: (1) multiple latent profiles of health-promoting lifestyles would be identified among community-dwelling older adults; and (2) Factors influencing different latent profiles of health-promoting lifestyles among community-dwelling older adults may vary.

## 2 Method

### 2.1 Study design and participants

This cross-sectional survey study was conducted from April 10, 2024, to July 18, 2024. A convenience sampling method was employed to recruit older adults from communities in Wuxing and Nanxun Districts, Huzhou City, Zhejiang, China. The inclusion criteria were: (1) age ≥60 years. (2) long-term residence in the community (≥ 6 months). (3) Clear consciousness, able to communicate effectively. The exclusion criteria were as follows: (1) Cognitive function screening using the Chinese version of the Mini-Mental State Examination(MMSE), which sets criteria for cognitive impairment based on a participant’s level of education: never educated ≤17, 1–6 years of schooling ≤20, and more than 6 years of education ≤24 (40). (2) a previous history of mental illness. (3) visual, hearing, and speech abnormalities that prevent normal communication. (4) suffering from severe physical diseases, such as heart failure or renal failure. (5) Inability to collaborate or early termination by the investigator.

Huzhou City, Zhejiang Province, is located in the eastern part of China. It has a relatively developed economy and has entered a stage of a deeply aging society. Under the promotion of local governments, the region is systematically building a multi-level and inclusive “senior care + enjoyment of old age” service system covering both urban and rural areas. Measures include “one household, one policy” home-based age-friendly renovations and the construction of a “15-minute senior care service circle”, providing a cutting-edge practical sample for similar regions. Therefore, this study, by analyzing the health-promoting lifestyles of older adults in the community of this region, aims to provide theoretical inspiration and empirical references for the formulation of health policies for older adults in regions with similar development levels.

### 2.2 Sample size

The cross-section sample size was calculated by the formula $n={\left(\frac{u_{\alpha /2}\sigma }{\delta }\right)}^{2}$ (41) assuming *α* = 0.05 and *δ* = 1.80. The pre-survey indicated that *σ* was 18.84. Given the sample missing and failure rates, the sample size was increased by 10.0%. This resulted in a required sample size of at least 463 cases for this study. In total, 520 questionnaires were distributed, and 503 were deemed valid, representing an effective recovery rate of 96.7%. This met the sample size requirement. The analysis employed listwise deletion to address missing values.

### 2.3 Measures

#### 2.3.1 Sociodemographic information questionnaire

Questionnaires included age, gender, number of chronic diseases, long-term medication categories, smartphone usage, marital status, residential status, number of children, monthly income, geriatric activities, household registration type, medical insurance types, and government subsidy.

#### 2.3.2 Health-promoting lifestyle profile-Chinese older adults (HPLP-CE)

Cao et al. revised the HPLP-CE scale based on the HPLP-IIC scale (42). This revised scale comprises 36 items across six dimensions: interpersonal support (6 items, e.g., Maintain meaningful and fulfilling interpersonal relationships), stress management (7 items, e.g., Respect my own accomplishments), self-actualization (5 items, e.g., Appreciate myself), Health Responsibility (10 items, e.g., Observe body monthly for physical changes), Physical Activity (4 items, e.g., Practice 15–20 min relaxation daily), and Nutrition (4 items, e.g., Eat three regular meals). Health-promoting lifestyle behavior was assessed using a 4-point Likert scale (Never = 1, Sometimes = 2, Often = 3, Routinely = 4), yielding scores ranging from 36 to 144, where higher scores indicate a healthier lifestyle. This scale demonstrates good reliability and validity among Chinese older adults. In this study, the Cronbach’s alpha coefficient for the total scale reached 0.94.

#### 2.3.3 Friend network

This study employed the friend network subscale of the revised Lubben Social Network Scale 6 (LSNS-6) (43), which has 3 items, each with a score range of 0–5 points (0 means no friends; 5 means >9 friends). The scale ranges from 0 to 15 points, with higher scores indicating a better relationship with a friend. A total score below 6 is indicative of friend isolation. The Cronbach’s *α* coefficient of the Friend Network Scale in this study was 0.93.

#### 2.3.4 Health risk perception

The health risk perception questionnaire developed by Zhou and Lin based on the health belief model was adopted (44). It includes eight items for the two dimensions of perceived susceptibility and perceived severity. The questionnaire uses a 5-point Likert scale ranging from 1 to 5 (1 = strongly disagree; 5 = strongly agree). The higher the score, the higher the perceived level of health risk. Cronbach’s *α* coefficient of this scale was 0.80 in this study.

### 2.4 Ethical considerations

The study adhered to the ethical principles of the Declaration of Helsinki and was approved by the Institutional Review Board of Hu Zhou University (Approval no. 2023-4-20) and the China Clinical Trial Registry (ChiCTR 2400082956).

### 2.5 Data collection

Before the investigation began, the interviewers received unified training and were able to provide consistent guiding language during the investigation. During the pre-survey, the interviewers found that the time it took for respondents to complete the questionnaires during the survey process was basically controlled within 10 to 15 min. During the formal investigation, the respondents were informed of the research purpose, content, and the benefits of participating in the project, and were also informed that they had the right to refuse to participate. After obtaining consent, the respondents signed the informed consent form. The investigation was conducted face-to-face. Respondents were encouraged to complete the questionnaire independently. For older adults with reading or vision difficulties, investigators provided assistance when necessary. Data collectors conduct on-site quality assessments of the completed questionnaires. Once they identify obvious missing responses or significant logical inconsistencies, they promptly verify with the respondents to ensure the accuracy and reliability of the data. After completing the questionnaire, each respondent was given a small gift worth 5 US dollars. On the basis of adopting the method of two-person data entry, 10% of the questionnaires were randomly selected for re-examination to ensure the accuracy of data entry.

### 2.6 Data analysis

The data were analyzed using SPSS 26.0 and Mplus 8.3. Firstly, all variables about the participants were subjected to a descriptive analysis. The measurement information was presented as a mean ± standard deviation, while the count information was represented by frequency and percentage. Secondly, latent profile analysis was conducted using Mplus 8.3. Each item of the HPLP scale for community-residing seniors was treated as an exogenous variable to construct a series of latent profile models. The initial assumption was complete independence among observed variables. The initial model started with one profile, gradually increasing the number of profiles (1–5). Models were estimated under the framework of maximum likelihood estimation (MLE); specifically, we adopted the maximum likelihood robust estimator (MLR) for model fitting. To reduce the risk of converging to local optima, we set 200 random starting points. Fit indices for each model were calculated, and model fit was evaluated based on information criteria, classification criteria, and likelihood ratio tests (45, 46). (1) The information criterion include the Akaike Information Criterion (AIC), Bayesian Information Criterion (BIC), and Sample-size Adjusted Bayesian Information Criterion (aBIC). These criteria evaluate the fitting effect, with smaller statistical values indicating better model fitting. (2) Classification criteria include the Entropy index to assess the accuracy of category classification, with a value ranging from 0 to 1. An entropy value ≥ 0.80 indicates that the model’s classification accuracy exceeds 90.0%. (3) Likelihood ratio test criteria include the Lo–Mendell–Rubin likelihood ratio test (LMRT) and Bootstrap Likelihood Ratio Test (BLRT). These tests compare the fitting differences of category models, with a *p* < 0.05 indicating that the k-profile model fits better than the k-1 profile model. The best profile model was selected, and each profile was named by combining the results of the three sets of evaluation indicators with the practical importance of the study and the number of samples in each profile (47).

Based on the optimal profile model of health-promoting lifestyles for community-dwelling older adults, statistical methods such as one-way ANOVA, chi-square test, and Fisher’s exact test were used to analyze differences in variables among different subgroups. For variables with significant differences, multiple comparisons were conducted using the Bonferroni correction. Subsequently, variables showing significant correlations in the univariate analysis were included in a multivariate logistic regression model, with stepwise selection applied for analysis, using *p* < 0.05 as the threshold for significance.

## 3 Results

### 3.1 Participants’ sociodemographic characteristics

The total number of older adults analyzed in this study was 503. The mean age of the participants was 69.38 ± 6.41 years, with males accounting for more than half of the total (*n* = 258, 51.3%). The smallest percentage of older individuals reported having four or more chronic diseases (*n* = 49, 9.7%) (Table 1).

**Table 1:** Characteristics of older people in the community (*n* = 503).

Variable	Classification	N	Percentage (%)	Mean ± SD
Age				69.38 ± 6.41
Gender	Male	258	51.3	
	Female	245	48.7	
Number of chronic diseases	0–1	354	70.4	
	2	106	21.1	
	≥3	43	8.5	
Long-term medication	0~1	349	69.4	
	2~3	105	20.9	
	≥4	49	9.7	
Smartphone usage	No	271	53.9	
	Yes	232	46.1	
Marital status	Married	426	84.7	
	Widowed/divorced/single	77	15.3	
Residential status	Alone	57	11.3	
	Living with children	91	18.1	
	Living with spouse	355	70.6	
Number of children	0	5	1.0	
	1	166	33.0	
	2	251	49.9	
	≥3	81	16.1	
Monthly income (yuan)	<1,000	189	37.6	
	1,001~3,000	183	36.4	
	>3,000	131	26.0	
Geriatric activities	Yes	188	37.4	
	No	315	62.6	
Household registration type	Urban	215	42.7	
	Rural	288	57.3	
Medical insurance	URRBMI	322	64.0	
	UEBMI	101	20.1	
	Other medical insurance	80	15.9	
Government subsidy	Enjoyment	45	8.9	
	Non-enjoyment	458	91.1	
Friend network				5.29 ± 3.79
Health risk perception				27.21 ± 4.22
Health-promoting lifestyle				88.09 ± 19.84

URRBMI, Urban–Rural Resident Basic Medical Insurance; UEBMI, Urban Employee Basic Medical Insurance.

### 3.2 Latent profile analysis of health-promoting lifestyle

**Figure 1:**
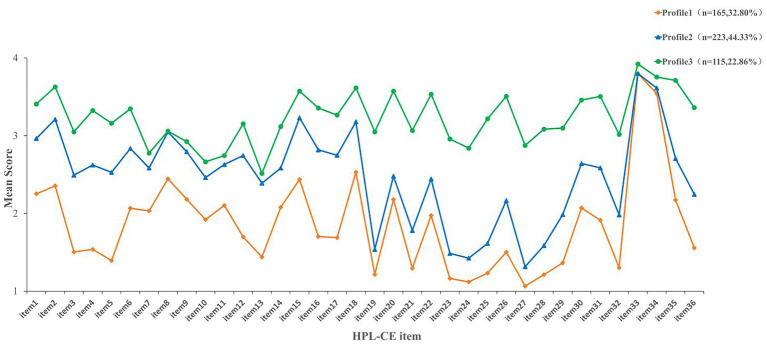
Three potential profiles of HPL-CE. The HPL-CE consists of 6 dimensions and 36 items. Dimension 1: Interpersonal support (IS) Item 1–Item 6 (6 items); Dimension 2: Stress management (SM) Item 7–Item 13 (7 items); Dimension 3: Self-actualization (SA) Item 14–Item 18 (5 items); Dimension 4: Health responsibility (HR), Item 19–Item 28 (10 items); Dimension 5: Physical activity (PA), Item 29 – Item 32 (4 items); Dimension 6: Nutrition (NT), Item 33–Item 36 (4 items).

The results of the model fit indices for the 1–5 latent profile models are shown in Table 2. (1) A comparison of AIC, BIC, and aBIC information criteria indicates a monotonic decrease in the three fit indices values as the number of classifications increases. (2) A comparison of the entropy values indicates that all models have high entropy values (> 0.80), indicating better differentiation between different groups in profiles 2, 3, 4, and 5 profile models. (3) A comparison of the likelihood ratio tests indicates that the LMRT and BLRT *p*-values are significant in the 3-profile model (*p* < 0.001), indicating that the 3-profile model is better than the 2-profile model. However, the 4-profile model and 5-profile model have LMRT and BLRT *p*-values that were not statistically significant (*p* > 0.05), so the 4-and 5-profile models were rejected. Based on a comprehensive evaluation of criteria and interpretability, the 3-profile model is deemed most suitable as the data model. Furthermore, the average posterior classification probabilities for the three-profile model were calculated as Profile 1 (98.3%), Profile 2 (96.8%), and Profile 3 (99.4%). These consistently high values indicate a high classification quality for the latent profile analysis, demonstrating that the three-profile solution exhibits excellent distinctiveness and classification accuracy. The specific information is shown in Table 3. Therefore, the 3-profile model was used as the optimal model for subsequent analyses (Figure 1; Supplementary Table S1).

**Table 2:** Latent profile model and fit information for health-promoting lifestyle (*n* = 503).

Model	Loglikelihood	AIC	BIC	aBIC	Entropy	LMRT	BLRT	Category probability (%)
						*p*-value		
1-Profile	−24283.74	48711.48	49015.36	48786.83	N/A	N/A	N/A	100.0
2-Profile	−21811.40	43840.79	44300.84	43954.86	0.99	<0.001	<0.001	74.8/25.3
**3-Profile**	**−20874.30**	**42040.60**	**42656.80**	**42193.39**	**0.95**	**<0.001**	**<0.001**	**32.8/44.3/22.9**
4-Profile	−20432.57	41231.13	42003.50	41422.64	0.97	0.658	<0.001	28.6/15.9/35.2/20.3
5-Profile	−20080.43	40600.85	41529.38	40831.08	0.98	0.215	<0.001	26.6/15.5/34.2/12.9/10.7

NA, Not appropriate; AIC, Akaike information criterion; BIC, Bayesian information criterion; aBIC, sample-size adjusted Bayesian Information Criterion; LMRT, Lo–Mendell–Rubin likelihood ratio test; BLRT, Bootstrap likelihood ratio test. The selected optimal model is shown in bold.

**Table 3:** Average posterior classification probability for the three latent classes.

Class	Profile 1(%)	Profile 2(%)	Profile 3(%)
Profile 1	**0.983**	0.017	0.000
Profile 2	0.024	**0.968**	0.008
Profile 3	0.000	0.006	**0.994**

Bolded values represent the average posterior classification probability for class membership.

### 3.3 Predictors of health-promoting lifestyle profiles

Table 4 presents a one-way analysis of the three subgroups regarding different characteristics. The results show that different HPL populations in terms of age, gender, number of chronic diseases, long-term medication types, smartphone usage, marital status, residence status, monthly income, participation in geriatric activities, household registration type, medical insurance, friend networks, and health risk perception. There are statistical differences in aspects (*p* < 0.05).

**Table 4:** Univariate analysis of potential profiles of health-promoting lifestyles in community-dwelling older adults.

Variable	Classification	Profile 1 (*n* = 165)	Profile 2 (*n* = 223)	Profile 3 (*n* = 115)	F/ $\chi^{2}$	*p*-value	$\omega^{2}$ /Cramer’s V
Age		71.95 ± 6.73	68.40 ± 5.71^*^	67.61 ± 6.15^*^	21.89^a^	**<0.001**	0.077
Gender					7.94^b^	**0.019**	0.126
	Male	96(58.2)	99(44.4)^*^	63(54.8)			
	Female	69(41.8)	124(55.6)^*^	52(45.2)			
Number of chronic diseases					25.40^b^	**<0.001**	0.159
	0–1	92(55.8)	174(78.0)^*^	88(76.5)^*^			
	2	52(31.5)	34(15.2)^*^	20(17.4)^*^			
	≥3	21(12.7)	15(6.7)	7(6.1)			
Long-term medication					30.52^b^	**<0.001**	0.174
	0~1	89(53.9)	169(75.8)^*^	91(79.1)^*^			
	2~3	56(33.9)	35(15.7)^*^	14(12.2)^*^			
	≥4	20(12.1)	19(8.5)	10(8.7)			
Smartphone usage					156.19^b^	**<0.001**	0.557
	Yes	18(10.9)	116(52.0)^*^	98(85.2)^*#^			
	No	147(89.1)	107(48.0)^*^	17(14.8)^*#^			
Marital status					25.50^b^	**<0.001**	0.225
	Married	121(73.3)	198(88.8)^*^	107(93.0)^*^			
	Widowed/divorced/single	44(26.7)	25(11.2)^*^	8(7.0)^*^			
Residential status					17.57^b^	**<0.001**	0.132
	Alone	31(18.8)	21(9.4)^*^	5(4.3)^*^			
	Live with children	23(13.9)	41(18.4)	27(23.5)			
	Live with spouse	111(67.3)	161(72.2)	83(72.2)			
Number of children					8.10^c^	0.204	0.098
	0	4(2.4)	1(0.4)	0(0.0)			
	1	61(37.0)	69(30.9)	36(31.3)			
	2	77(46.7)	119(53.4)	55(47.8)			
	≥3	23(13.9)	34(15.2)	24(20.9)			
Monthly income (yuan)					109.00^b^	**<0.001**	0.329
	<1,000	102(61.8)	71(31.8)^*^	16(13.9)^*#^			
	1,001 ~ 3,000	56(33.9)	89(39.9)	38(33.0)			
	>3,000	7(4.2)	63(28.3)^*^	61(53.0)^*#^			
Geriatric activities					92.80^b^	**<0.001**	0.430
	Yes	26(15.8)	79(35.4)^*^	83(72.2)^*#^			
	No	139(84.2)	144(64.6)^*^	32(27.8)^*#^			
Household registration					69.25^b^	**<0.001**	0.371
	Urban	40(24.2)	90(40.4)^*^	85(73.9)^*#^			
	Rural	125(75.8)	133(59.6)^*^	30(26.1)^*#^			
Medical insurance					113.64^b^	**<0.001**	0.336
	URRBMI	110(66.7)	162(72.6)	50(43.5)^*^			
	UEBMI	10(6.1)	31(13.9)^*^	60(52.2)^*#^			
	Other	45(27.3)	30(13.5)^*^	5(4.3)^*#^			
Government subsidy					2.70^b^	0.259	0.073
	Enjoyment	10(6.1)	22(9.9)	13(11.3)			
	Non-enjoyment	155(93.9)	201(90.1)	102(88.7)			
Friend network		2.35 ± 2.46	6.28 ± 3.35^*^	7.57 ± 3.61^*#^	135.77^a^	**<0.001**	0.308
Health risk perception		25.70 ± 4.40	28.07 ± 3.97^*^	27.73 ± 3.83^*^	15.71^a^	**<0.001**	0.060

URRBMI, Urban–Rural Resident Basic Medical Insurance; UEBMI, Urban Employee Basic Medical Insurance. The letter “a” indicates the use of one-way ANOVA, letter “b” indicates the use of chi-square test, letter “c” indicates the use of Fisher’s exact test, and post hoc test uses Bonferroni method. * indicates comparison with Profile 1, *p* < 0.05; # indicates comparison with Profile 2, *p* < 0.05. The effect size of one-way ANOVA is measured using ω^2^, with a criterion of 0.01 indicating a small effect, 0.06 indicating a medium effect, and 0.14 indicating a large effect; The effect size of $\chi^{2}$ /Fisher’s exact test is measured using Cramer’s V, with a criterion of 0.1 indicating a small effect, 0.3 indicating a medium effect, and 0.5 indicating a large effect. Significant factors are in bold.

Multivariate logistic regression analysis was conducted with the HPL profile as the dependent variable. Variables with *p* < 0.05 in the univariate analysis were selected as independent variables (Table 5). The variance inflation factor (VIF) was used to diagnose collinearity for all independent variables, and all VIF values were below 10 (Supplementary Table S2) (48), indicating that there was no significant multicollinearity problem among the variables. Based on the model fit information of the multivariable logistic regression, the model exhibited overall statistical significance (Pseudo-*R*^2^ = 0.571, χ^2^ = 425.846, *p* < 0.001). The results indicated that gender, the number of chronic diseases, smartphone usage, residential status, monthly income, geriatric activities, household registration type, medical insurance, friend networks, and health risk perception were related to health-promoting lifestyles of older adults in the community (*p* < 0.05).

**Table 5:** Multiple logistic regression of latent profiles of health-promoting lifestyle in community-dwelling older adults.

Variable	Classification	Profile 1(Ref) vs Profile 2			Profile 1 (Ref) vs Profile3			Profile 2 (Ref) vs Profile3		
		OR	95% CI	*p*-value	OR	95% CI	*p*-value	OR	95% CI	*p*-value
Gender (Ref: male)	Female	2.127	1.187–3.811	**0.011**	1.771	0.819–3.832	0.147	0.833	0.465–1.492	0.539
Number of chronic diseases (Ref:0–1)	>3	0.520	0.206–1.312	0.166	0.839	0.223–3.157	0.796	1.614	0.501–5.201	0.422
	2	0.342	0.168–0.695	**0.003**	0.312	0.117–0.834	**0.020**	0.915	0.413–2.028	0.826
Smartphone usage (Ref: no)	Yes	4.582	2.332–9.004	**<0.001**	17.148	7.068–41.603	**<0.001**	3.742	1.887–7.423	**<0.001**
Residential status (Ref: live alone)	Live with children	5.730	1.793–18.316	**0.003**	8.393	1.696–41.532	**0.009**	1.465	0.401–5.345	0.563
	Live with spouse	3.109	1.157–8.355	**0.025**	2.738	0.671–11.169	0.160	0.881	0.274–2.825	0.831
Monthly income(yuan) (Ref:<1,000)	>3,000	8.853	2.514–31.179	**<0.001**	7.365	1.685–32.188	**0.008**	0.832	0.333–2.081	0.694
	1,000–3,000	1.390	0.764–2.532	0.281	1.393	0.557–3.484	0.479	1.002	0.453–2.213	0.996
Geriatric activities (Ref: no)	Yes	2.342	1.177–4.661	**0.015**	8.356	3.604–19.375	**<0.001**	3.569	1.978–6.438	**<0.001**
Household registration (Ref: rural)	Urban	0.910	0.479–1.729	0.773	2.148	0.938–4.917	0.070	2.361	1.270–4.390	**0.007**
Medical insurance (Ref: other)	URRBMI	2.152	1.018–4.549	**0.045**	3.281	0.893–12.056	0.074	1.524	0.477–4.874	0.477
	UEBMI	0.925	0.260–3.290	0.904	5.506	1.080–28.054	**0.040**	5.952	1.803–19.651	**0.003**
Friend network		1.395	1.265–1.539	**<0.001**	1.434	1.267–1.623	**<0.001**	1.028	0.943–1.121	0.532
Health risk perception		1.122	1.042–1.208	**0.002**	1.064	0.971–1.167	0.182	0.949	0.887–1.014	0.122

Ref: Reference. OR: Odds ratio; 95% CI: 95% Confidence Interval. Profile 1: low health-promoting lifestyle group; Profile 2: moderate health-promoting lifestyle—low health responsibility group; Profile 3: high health-promoting lifestyle—low stress management group.χ^2^ = 425.846, *p <* 0.001, Nagelkerke’s Pseudo-R^2^ = 0.571, Significant factors are in bold.

Taking Profile1 as the reference group, female (OR = 2.127, 95%, CI: 1.187–3.811), smartphone usage (OR = 4.582, 95% CI: 2.332–9.004), living with children (OR = 5.730, 95% CI: 1.793–18.316), living with spouse (OR = 3.109, 95% CI: 1.157–8.355), monthly income greater than 3,000 RMB (OR = 8.853, 95% CI: 2.514–31.179), participation in community geriatric activities (OR = 2.342, 95% CI: 1.177–4.661), having URRBMI (OR = 2.152, 95% CI: 1.018–4.549), friend network (OR = 1.395, 95% CI: 1.265–1.539), and health risk perception (OR = 1.122, 95% CI: 1.042–1.208) were more likely to be associated with the moderate health—promoting lifestyle—low health responsibility group. The number of chronic diseases was 2 (OR = 0.342, 95% CI: 0.168–0.695) is associated with a higher tendency to belong to a low health-promoting lifestyle group.

Taking Profile1 as the reference group, smartphone usage (OR = 17.148, 95%CI: 7.068–41.603), living with children (OR = 8.393, 95%CI: 1.696–41.532), monthly income > 3,000 yuan (OR = 7.365, 95%CI: 1.685–32.188), participation in community geriatric activities (OR = 8.356, 95%CI: 3.604–19.375), having UEBMI (OR = 5.506, 95%CI: 1.080–28.054), friend network (OR = 1.434, 95%CI: 1.267–1.623) were more likely to favor the high health-promoting lifestyle—low stress management group. The number of chronic diseases was 2 (OR = 0.312, 95%CI: 0.117–0.834) is associated with a higher tendency to belong to a low health-promoting lifestyle group.

Taking Profile2 as the reference group, smartphone usage (OR = 3.742, 95%CI: 1.887–7.423), participation in community geriatric activities (OR = 3.569, 95%CI: 1.978–6.438), urban household registration type (OR = 2.361, 95%CI: 1.270–4.390), having UEBMI (OR = 5.952, 95%CI: 1.803–19.651) were more likely to be in the high health-promoting lifestyle-low stress management group.

## 4 Discussion

This study employed latent profile analysis to explore latent subgroups of HPL among community-dwelling older adults and to investigate the factors influencing these subgroups. Based on comparative model fit results, interpretability, and practical significance, our study reveals that HPL among older adults living in the community can be categorized into three subgroups: low health-promoting lifestyle, moderate health-promoting lifestyle-low health responsibility, and high health-promoting lifestyle-low stress management. This finding offers a new perspective in the field of health-promoting lifestyles for older adults, emphasizing the heterogeneity of such lifestyles within this population.

The low health-promoting lifestyle group accounted for 32.8% of participants, with scores ranging from 1.07 to 3.80 and an average score of 1.86. Compared to the other two subgroups, this group scored lowest across all items, indicating an overall low level of HPL and indicating markedly insufficient engagement in health behaviors. These older adults appear to experience cumulative disadvantages, including poor physical functioning, limited health literacy, reduced social participation, and socioeconomic constraints, which together may weaken motivation and capacity to pursue healthy lifestyles (49, 50). Consequently, this subgroup should be prioritized in community-based aging interventions. A coordinated support model integrating family doctors, community health workers, and family caregivers is recommended to ensure continuous monitoring, tailored counseling, and reinforcement of basic behaviors such as regular routines and low-salt diets. For older adults with functional limitations, tailored care and rehabilitation interventions should be prioritized, alongside strengthened family involvement, community-based support, and psychological care. In addition, creating age-friendly opportunities for social participation is essential to enhance health engagement, improve quality of life, and sustain long-term health behaviors (51).

In the moderate health-promoting lifestyle—low health responsibility group, this group accounted for 44.3%, with scores ranging from 1.32 to 3.80 (mean = 2.48), and the overall HPL level was moderate. However, the performance of this subgroup in the “health responsibility” dimension was significantly weaker, with scores ranging from 1.32 to 2.48 (mean = 1.78), especially reflected in the lower scores for “participating in health education activities” and “engaging in vigorous exercise”. This group of characteristics is in line with the Pender health promotion theory model (30), reflecting that older individuals have selective cognition of health behaviors, and they prefer health behaviors that can enhance autonomy and bring positive experiences, while they are less likely to choose to face health behaviors such as active monitoring of vital signs and seeking professional health guidance. This leads to low self-efficacy and high perceptual impairment of their health responsibilities. This pattern reflects insufficient proactive health awareness, consistent with evidence that higher health consciousness facilitates active self-management and adoption of healthy behaviors (52). Limited access to public health resources may further hinder health responsibility in this population (53). Targeted efforts should therefore emphasize strengthening self-management awareness and resource access, including regular screening and brief counseling delivered by family doctors and community nurses, alongside improved accessibility to community exercise and educational resources, to facilitate a shift from passive care receipt to active health management and enhance HPL in this subgroup (54).

The high health-promoting lifestyle–low stress-management subgroup accounted for 22.9% of the sample, with a score range of 2.52–3.92 (mean = 3.23), showing a high level of health promotion as a whole, but the stress management dimension was relatively weak, with a score range of 2.52–3.15 (mean = 2.83). This group scored relatively low in items such as “Appropriately responding to unreasonable Demands” and “Identifying sources of life stress”, suggesting that their ability to cope with stress is limited. This group indicates A strong motivation for a health-promoting lifestyle but relatively insufficient stress regulation, which is in line with the perspective of the interaction between the environment and society emphasized by socio-ecological theory (55). Analysis shows that although effective support has been provided in promoting the physical health of the aging population from the individual to the macro level, shaping the characteristics of a high-level health-promoting lifestyle, factors such as the lack of deep emotional interaction at the interpersonal level, the absence of psychological support norms at the community level, and insufficient investment in mental health resources for older adults at the social level. This led to weak stress management in this group (56, 57). Inadequate stress management can erode confidence when facing multiple stressors, hinder adherence to health-promoting behaviors, and reinforce negative self-appraisals (58). Chronic stress, especially from other-focused burdens, has also been linked to accelerated cognitive decline (59). Therefore, brief stress screening should be integrated into routine health assessments, and combined with concise psychoeducation, mindfulness and breathing exercises, structured social participation, essential counseling, and online resources, as well as emotional support from family and peers, to systematically identify stressors, strengthen coping capacity, and sustain health-promoting behaviors (60).

This study found that gender, the number of chronic diseases, smartphone usage, residential status, monthly income, geriatric activities, household registration type, medical insurance, friend network, and health risk perception are significantly associated with different characteristics of health-promoting lifestyles.

Regarding gender differences, older women were more likely than men to be classified into the moderate health-promoting lifestyle–low health-responsibility group. Although frequent participation in social interactions may provide greater exposure to health information (33), their lower health responsibility scores may be attributed to substantial family obligations, such as childcare and household tasks, which can limit time and attention devoted to self-care and weaken personal health responsibility (61).

In terms of the number of chronic diseases, older adults in the community with two types of chronic diseases are more inclined to the group with the low health-promoting lifestyle. Xu et al. (56) research indicates that the number of chronic diseases is regarded as an important influencing factor for the health-promoting lifestyle of older adults. Most older adults are generally troubled by the coexistence of chronic diseases, which not only increases the burden of medical treatment, medication, and economy (62), but also leads to a decline in the health-related quality of life of older people (63). Therefore, primary health care workers need to pay attention to the management of chronic diseases among older adults, attach importance to enhancing their proactive health awareness, and help them establish and maintain a healthy lifestyle.

Compared with nonusers, usage smartphone older adults were more likely to fall into the moderate health-promoting lifestyle-low health responsibility group and the high health-promoting lifestyle–low stress management group. This pattern aligns with the knowledge–attitude–practice framework (64), whereby smartphones facilitate access to health information and enhance health awareness, promoting proactive health behaviors. Evidence indicates that smartphone applications can digitally assess lifestyle and cognition in later life and support behavior change (65). Nevertheless, misinformation and a persistent digital divide remain barriers, particularly for those living alone (66). Strengthening e-health literacy among older adults, with targeted support for solo dwellers, is essential to ensure accurate health information use and adoption of appropriate behaviors (67, 68), thereby advancing equitable digital participation in healthy aging.

Compared with older adults living alone, those living with their children are more likely to be categorized into the moderate health-promoting lifestyle–low health-responsibility group and the high health-promoting lifestyle–low stress management group. Older adults living with their spouses tend to fall into the moderate health-promoting lifestyle–low health-responsibility group, a finding that aligns with prior research (18). For older adults cohabiting with their children, intergenerational interactions facilitate access to health-related information and daily monitoring, thereby promoting greater engagement in physical activity and social participation (36). In addition, the socio-emotional choice theory posits that the older adults place greater emphasis on social relationships with profound emotional significance. Consequently, the companionship of spouses and children exerts a significant protective influence on their health (69). Collectively, these mechanisms contribute to a healthier lifestyle among older adults co-residing with their spouses or children. Therefore, the role of family support ought to be further leveraged in health promotion, and the establishment of a family-community collaborative model should be advanced to offer alternative social support and psychological care for the older adults living alone, thereby promoting health equity within the older population.

Older adults with higher monthly incomes were more likely to fall into the moderate health-promoting lifestyle–low health-responsibility group and the high health-promoting lifestyle–low stress-management group, consistent with previous evidence (53, 70). Greater financial resources enable access to preventive check-ups, structured exercise, and wellness counseling, facilitating healthier lifestyle adoption (71, 72). These findings highlight the influence of socioeconomic inequality on older adults’ capacity to sustain health-promoting behaviors. Moreover, income level has been shown to moderate the relationship between social participation and loneliness (73). Accordingly, targeted financial support for low- and middle-income older adults may reduce barriers to participation in social and health-promoting activities, potentially enhancing behavioral adherence and mitigating psychosocial risks.

Notably, the older adults who participated in geriatric activities were more likely to belong to the moderate health-promoting lifestyle-low health responsibility group and the high health-promoting lifestyle-low stress management group. This finding is consistent with the study conducted by Zhang et al. (53), which indicates that geriatric activities offer a platform for active interaction and information exchange among older adults, thus facilitating healthier behavior patterns. The World Health Organization identifies social participation as one of the three pillars of active aging (74). Older adults involved in community programs are more likely to receive peer support, reduce social isolation, and enhance their sense of belonging, thereby strengthening motivation and capacity to adopt health-promoting behaviors (75). In conclusion, these findings underscore the importance of strengthening community-based participation mechanisms and activity provision to promote sustained healthy behaviors among older adults, and future efforts should optimize community resource allocation and program models to maximize reach and impact.

Regarding the household registration type, older individuals residing in urban communities were inclined to belong to the high health-promoting lifestyle-low stress management group. Over 70% of older adults in the high health-promoting lifestyle-low stress management group were from urban communities. Han et al. (18) indicated that there might be disparities in the health-promoting lifestyle of older people between urban and rural areas, which could be associated with the variations in resource conditions, social environment, and cognitive level between these two areas. Older adults in urban communities are more likely to have access to well-developed aging facilities, convenient medical and health services, and rich social support networks, which facilitate older individuals in maintaining healthy behaviors. Therefore, when formulating strategies for promoting the health of the aging population in the future, attention should be paid to the differences between urban and rural areas, and emphasis should be placed on exploring the path of health-promoting lifestyle for rural-dwelling older adults.

In terms of health insurance, older adults enrolled in the URRBMI were more likely to be categorized into the moderate health-promoting lifestyle–low health responsibility group. The continuous expansion of URRBMI has improved healthcare accessibility and reduced financial burdens among older adults. Notably, those covered by the UEBMI tended to be in the highly health-promoting lifestyle-low stress management group. This may be due to its higher reimbursement rate and more comprehensive benefits. Therefore, strengthening the coordination between health insurance policies and health education, extending insurance coverage for chronic disease management, and promoting community-based health interventions may further encourage healthy lifestyles among older adults.

This study demonstrates that disparities exist between the friend network and older adults’ health-promoting lifestyle categories in the community, which aligns with the findings of previous research (76). Older adults with a higher level of friend network are likely to exhibit a more pronounced health-promoting lifestyle. They are more prone to being classified into the “moderate health-promoting lifestyle–low health responsibility” and “high health-promoting lifestyle–low stress management” groups. It is noteworthy that the friend network may exert a more significant influence on promoting the health behavior of older adults compared to the family network (32). This might be attributed to the fact that information exchange and emotional support among peers are more conducive to the formation and maintenance of healthy behavior patterns (77, 78). Therefore, community buddy activities and friend support mechanisms should be enhanced to facilitate the dissemination of health information and the adoption of healthy behaviors.

Finally, health risk perceptions differed among different community older adults health-promoting lifestyle categories, with older adults with high health risk perceptions tending to the moderate health-promoting lifestyle -low health responsibility group, which is consistent with Ferrer’s findings (79). Ferrer highlighted the importance of health risk perception in determining health behavior. Additionally, previous studies have demonstrated that the health risk perception among older people is associated with their information-seeking behavior and active healthy lifestyle (80). Therefore, enhancing the older adults’ awareness of health risks is of crucial importance for guiding them to adopt a comprehensive healthy lifestyle.

This study is subject to several limitations. First of all, it should be pointed out that the sample of this study was only selected from the community residents of a certain city in Zhejiang Province, China, and a convenience sampling method was used, which may limit the representativeness of the sample. The research results reflect, to some extent, the situation of older residents in the communities of relatively developed areas in East China. However, caution should be exercised when extending them to different areas with less developed economies. It is suggested that future research adopt a multi-center and large-sample design scheme, covering groups from different regions and socio-economic backgrounds, in order to enhance the representativeness of the research results.

Secondly, the assessment of health-promoting lifestyles relies on self-reporting and a face-to-face interview format, which may introduce social desirability bias, recall bias, and minor interviewer bias, potentially affecting the accuracy of the study conclusions. Future research should incorporate objective physical measurement indicators (e.g., frailty, mobility) to further enhance the study’s accuracy.

Finally, as a cross-sectional study, this research cannot infer causal relationships or the mechanisms of interaction between variables. Therefore, future studies are advised to employ multi-level analysis from an intersectional perspective to elucidate the underlying mechanisms of various factors or to conduct longitudinal or interventional studies to clarify causal relationships.

## 5 Conclusion

In summary, based on the latent profile analysis technique, three subgroups of community-residing older adults with health-promoting lifestyles were identified: low health-promoting lifestyle group, moderate health-promoting lifestyle-low health responsibility group, and high health-promoting lifestyle-low stress management group. The diverse needs of the older population within communities provide a reference basis for formulating aging policies. In primary healthcare, this assists medical professionals in rapidly stratifying patients based on subgroup characteristics, designing community health promotion plans tailored to the specific needs of different subgroups, and optimizing resource allocation. Meanwhile, we identified that gender, the number of chronic diseases, smartphone use, residential status, monthly income, participation in geriatric activities, household registration type, medical insurance, friend networks, and health risk perception are significantly associated with different characteristics of health-promoting lifestyles. Therefore, future research should further explore the potential mechanisms underlying these key factors to inform the development of cost-effective interventions. This may help individuals enhance their levels of health-promoting lifestyles and improve their social well-being.

## Data Availability

The raw data supporting the conclusions of this article will be made available by the authors, without undue reservation.

## References

[1] The State Council Information Office SCIO press conference on China's economic performance in 2023. (2024). Available online at: http://english.scio.gov.cn/pressroom/2024-01/26/content_116967913.htm (Accessed January 14, 2024).

[2] United Nations Department of Economic and Social Affairs, Population Division. World Population Prospects 2024. (2024). Available online at: https://population.un.org/wpp/ (Accessed July 24, 2024).

[3] Xi JY Zhao JG Li XQ Yan B Bai JJ Xiang YN . Quantifying the loss of healthy life expectancy due to population ageing: health benefit estimation from a global perspective. BMJ Glob Health. (2025) 10:e018194. doi: 10.1136/bmjgh-2024-018194, 40341142 PMC12060892

[4] Khan HTA Addo KM Findlay H. Public health challenges and responses to the growing ageing populations. Public Health Challenges. (2024) 3:e213. doi: 10.1002/puh2.213, 40496520 PMC12039680

[5] Beard JR Officer A De CIA Sadana R Pot AM Michel JP . The world report on ageing and health: a policy framework for healthy ageing. Lancet. (2016) 387:2145–54. doi: 10.1016/S0140-6736(15)00516-4 26520231 PMC4848186

[6] eBioMedicine. Healthy ageing begins with a healthy lifestyle. EBioMedicine. (2023) 89:104528. doi: 10.1016/j.ebiom.2023.104528, 36907646 PMC10025755

[7] Visser M Wijnhoven HAH Comijs HC Thomése FGCF Twisk JWR Deeg DJH. A healthy lifestyle in old age and prospective change in four domains of functioning. J Aging Health. (2019) 31:1297–314. doi: 10.1177/0898264318774430, 29809092 PMC7322976

[8] Rizzuto D Orsini N Qiu C Wang HX Fratiglioni L. Lifestyle, social factors, and survival after age 75: population based study. BMJ. (2012) 345:e5568. doi: 10.1136/bmj.e5568, 22936786 PMC3431442

[9] Alinejad N Khosromanesh F Bijani M Taghinezhad A Khiyali Z Dehghan A. Spiritual well-being, resilience, and health-promoting lifestyle among older adult hypertensive patients: a cross-sectional study. BMC Geriatr. (2025) 25:265. doi: 10.1186/s12877-025-05877-x, 40269763 PMC12016123

[10] Walker SN Sechrist KR Pender NJ. The health-promoting lifestyle profile: development and psychometric characteristics. Nurs Res. (1987) 36:76–81. 3644262

[11] Wang L Wang Y Luo Y Li Y Li J. The mediating and moderating effect of health-promoting lifestyle on frailty and depressive symptoms for Chinese community-dwelling older adults: a cross-sectional study. J Affect Disord. (2024) 361:91–6. doi: 10.1016/j.jad.2024.06.011, 38857627

[12] Leung YS Lee JJW Lai MMP Kwok CKM Chong KC. Association between obesity, common chronic diseases and health promoting lifestyle profiles in Hong Kong adults: a cross-sectional study. BMC Public Health. (2020) 20:1624. doi: 10.1186/s12889-020-09726-x, 33115451 PMC7594285

[13] Navasca SB Lipardo D. Assessing the quality of life of the llocano older adults during COVID-19 pandemic: a cross-sectional study on the association between health-promoting behaviors and quality of life, and the moderating effect of socio-demographic profile. J Cross Cult Gerontol. (2024) 40:77–92. doi: 10.1007/s10823-024-09519-4, 39671138

[14] Angelsen A Nakrem S Zotcheva E Strand BH Strand LB. Health-promoting behaviors in older adulthood and intrinsic capacity 10 years later: the HUNT study. BMC Public Health. (2024) 24:284. doi: 10.1186/s12889-024-17840-3, 38267907 PMC10809656

[15] Wu Y Su B Chen C Zhao Y Zhong P Zheng X. Urban-rural disparities in the prevalence and trends of depressive symptoms among Chinese elderly and their associated factors. J Affect Disord. (2023) 340:258–68. doi: 10.1016/j.jad.2023.07.117, 37536424

[16] Su H Xu L Yu H Zhou Y Li Y. Social isolation and intrinsic capacity among left-behind older adults in rural China: the chain mediating effect of perceived stress and health-promoting behavior. Front Public Health. (2023) 11:1155999. doi: 10.3389/fpubh.2023.1155999, 37033030 PMC10080141

[17] Zhang X Kamin ST Liu S Fung HH Lang FR. Negative self-perception of aging and mortality in very old Chinese adults: the mediation role of healthy lifestyle. J Gerontol B Psychol Sci Soc Sci. (2020) 75:1001–9. doi: 10.1093/geronb/gby136, 30445618

[18] Han Y Xing F Huang J Wang M. Associated factors of health-promoting lifestyle of the elderly based on the theory of social ecosystem. Aten Primaria. (2023) 55:102679. doi: 10.1016/j.aprim.2023.102679, 37295306 PMC10272280

[19] Chang H Wang X Wang Z. Association between social capital and health-promoting lifestyle among empty nesters: the mediating role of sense of coherence. Geriatr Nurs. (2023) 53:96–101. doi: 10.1016/j.gerinurse.2023.07.006, 37473467

[20] Sok SR Cheon BK Gu MK Kim OS. Comparisons of health promoting behavior, depression, and life satisfaction between older adults in rural areas in South Korea living in group homes and at home. J Nurs Res. (2019) 27:e21. doi: 10.1097/JNR.0000000000000290, 30289790 PMC6553957

[21] Goller M Kyndt E Paloniemi S Damşa C. Methods for researching professional learning and development: challenges, applications and empirical illustrations. Cham: Springer International Publishing (2022).

[22] Yin K Peng J Zhang J. The application of latent profile analysis in organizational behavior research. Adv Psychol Sci. (2020) 28:1056–70. doi: 10.3724/SP.J.1042.2020.01056

[23] Collins LM Lanza ST. Latent class and latent transition analysis: With applications in the social, behavioral, and health sciences. Hoboken, NJ: John Wiley & Sons (2013).

[24] Koç KK Korkmaz Aslan G. Older people's perception and experience regarding health promotion in Turkey: a qualitative study. Clin Nurs Res. (2023) 32:850–60. doi: 10.1177/10547738221146415, 36625249

[25] Son EH Wallen GR Flynn S Lee LJ. Patterns of health-promoting behaviors and associated factors in family caregivers of people receiving cancer treatment: a latent class profile analysis. Psychooncology. (2023) 32:1038–47. doi: 10.1002/pon.6145, 37157152 PMC10590684

[26] Bishop NJ Zuniga KE Ramirez CM. Latent profile analysis of dietary intake in a community-dwelling sample of older Americans. Public Health Nutr. (2020) 23:243–53. doi: 10.1017/S1368980019001496, 31248470 PMC10200398

[27] Zhang Y Yao L Chen L Zhong W Li J Xu L . Longitudinal relationship between 24-hour movement behavior patterns and physical function and quality of life after stroke: a latent transition analysis. Int J Behav Nutr Phys Act. (2024) 21:141. doi: 10.1186/s12966-024-01689-1, 39696459 PMC11656942

[28] Liu Y Li B Liu L Chen X Liu W Yao M . Mental health literacy and its relationship with health-promoting behaviors of community-dwelling older adults: a latent profile analysis. Geriatr Nurs. (2025) 62:123–30. doi: 10.1016/j.gerinurse.2025.01.015, 39892328

[29] Luo D Yu S Wang J Zhu Y Yang L Bai R . Social participation of community-dwelling older adults in western China: a latent profile analysis. Front Public Health. (2022) 10:874204. doi: 10.3389/fpubh.2022.874204, 36081484 PMC9446436

[30] Chen HH Hsieh PL. Applying the Pender's health promotion model to identify the factors related to older adults' participation in community-based health promotion activities. Int J Environ Res Public Health. (2021) 18:9985. doi: 10.3390/ijerph18199985, 34639294 PMC8508522

[31] Guo L Liu Y Zhu Y Wei M. Identification of health behaviour clusters among people at high risk of stroke: a latent class profile analysis. J Adv Nurs. (2020) 76:3039–47. doi: 10.1111/jan.14523, 32888193

[32] Wu F Sheng Y. Social isolation and health-promoting behaviors among older adults living with different health statuses: a cross-sectional study. Int J Nurs Sci. (2021) 8:304–9. doi: 10.1016/j.ijnss.2021.05.007, 34307779 PMC8283714

[33] Oh J. Factors affecting health promoting behavior among older women in Korea: a structural equation model. Health Promot Int. (2021) 36:924–32. doi: 10.1093/heapro/daaa117, 33236075

[34] Zhou C Zheng W Yuan Q. Influence of health promoting lifestyle on health management intentions and behaviors among Chinese residents under the integrated healthcare system. PLoS One. (2022) 17:e0263004. doi: 10.1371/journalpone.0263004 35077472 PMC8789132

[35] Huang L Li H Liu H Tian H Luo H Wu J . Socioecological influencers of health-promoting lifestyles in Chinese: a preliminary survey using convenient samples. Front Public Health. (2024) 11:1309824. doi: 10.3389/fpubh.2023.1309824, 38259776 PMC10800470

[36] He L Xing X. Analysis of health promoting lifestyle of the elderlies and its related influencing factors. Guangdong Med J. (2021) 42:992–5. doi: 10.13820/j.cnki.gdyx.20211398

[37] Yun JY Yun YH. Health-promoting behavior to enhance perceived meaning and control of life in chronic disease patients with role limitations and depressive symptoms: a network approach. Sci Rep. (2023) 13:4848. doi: 10.1038/s41598-023-31867-3, 36964273 PMC10039031

[38] Wu F Sheng Y. Social support network, social support, self-efficacy, health-promoting behavior and healthy aging among older adults: a pathway analysis. Arch Gerontol Geriatr. (2019) 85:103934. doi: 10.1016/j.archger.2019.103934, 31466024

[39] Hareru HE Mamo TT Abebe M Debela BG. Health-promoting behavior and its determinants towards non-communicable diseases among adult residents of the Gedeo zone, South Ethiopia: the application of the health belief model. Front Public Health. (2024) 12:1453281. doi: 10.3389/fpubh.2024.1453281, 39324155 PMC11423864

[40] Huang X Deng J Liu W. Sex differences in cognitive function among Chinese older adults using data from the Chinese longitudinal healthy longevity survey: a cross-sectional study. Front Public Health. (2023) 11:1182268. doi: 10.3389/fpubh.2023.1182268, 37457255 PMC10343959

[41] Sun ZQ Xu YY. Medical statistics. Beijing: People's Medical Publishing House (2020).

[42] Cao WJ Chen CS Hua Y Li YM Xu YY Hua QZ. Factor analysis of a health-promoting lifestyle profile (HPLP): application to older adults in mainland China. Arch Gerontol Geriatr. (2012) 55:632–8. doi: 10.1016/j.archger.2012.07.003, 22854282

[43] Lubben J Blozik E Gillmann G Iliffe S von Renteln Kruse W Beck JC . Performance of an abbreviated version of the Lubben social network scale among three European community-dwelling older adult populations. Gerontologist. (2006) 46:503–13. doi: 10.1093/geront/46.4.503 16921004

[44] Zhou M Lin M. The positive-sum game between risk perception and self-efficacy: a study on the influencing factors of health information seeking behavior based on ELM model. BM (2020) 178:38–55. doi: 10.20050/j.cnki.xwdx.2020.09.006

[45] Peugh J Fan X. Modeling unobserved heterogeneity using latent profile analysis: a Monte Carlo simulation. Struct Equ Modeling. (2013) 20:616–39. doi: 10.1080/10705511.2013.824780

[46] Aflaki K Vigod S Ray JG. Part II: a step-by-step guide to latent class analysis. J Clin Epidemiol. (2023) 159:348–51. doi: 10.1016/j.jclinepi.2023.05.025, 37286148

[47] Sinha P Calfee CS Delucchi KL. Practitioner's guide to latent class analysis: methodological considerations and common pitfalls. Crit Care Med. (2021) 49:e63–79. doi: 10.1097/ccm.0000000000004710, 33165028 PMC7746621

[48] Dormann CF Elith J Bacher S Buchmann C Carl G Carré G . Collinearity: a review of methods for dealing with collinearity and simulation studies to evaluate its performance. Ecography. (2013) 36:27–46. doi: 10.1111/j.1600-0587.2012.07348.x

[49] Chen L Gong Y Yuan L. Health behaviour and its determinants in elderly patients with chronic diseases: evidence from Jiangsu Province, China. BMC Geriatr. (2022) 22:297. doi: 10.1186/s12877-022-03010-w, 35392819 PMC8988547

[50] Darabi F Ziapour A Janjani P Motevaseli S Rostami F. A cross-sectional study of the relationship between health literacy and health-promoting lifestyles in patients with hypertension in Northwest Iran. BMC Primary Care. (2025) 26:117. doi: 10.1186/s12875-025-02819-9, 40247154 PMC12004634

[51] Orhan İ Yağmur Y. The effect of motivational interviewing on healthy lifestyle behaviors and quality of life in on menopausal women: a pilot randomized controlled trial. BMC Womens Health. (2025) 25:306. doi: 10.1186/s12905-025-03807-y, 40611241 PMC12231738

[52] Zadworna M. Healthy aging and the University of the Third age - health behavior and subjective health outcomes in older adults. Arch Gerontol Geriatr. (2020) 90:104126. doi: 10.1016/j.archger.2020.104126, 32512462

[53] Zhang C Zhu R Lu J Xue Y Hou L Li M . Health promoting lifestyles and influencing factors among empty nesters and non-empty nesters in Taiyuan, China: a cross-sectional study. Health Qual Life Outcomes. (2018) 16:103. doi: 10.1186/s12955-018-0936-5, 29801495 PMC5970479

[54] Ashrafi E Izadi B Safari O Hassankiadeh RF Mansourian M. The effect of multimodal educational interventions on improving the lifestyle of the elderly: a quasi-experimental study. BMC Public Health. (2025) 25:2795. doi: 10.1186/s12889-025-24048-6, 40819050 PMC12357333

[55] Mcleroy KR Bibeau D Steckler A Glanz K. An ecological perspective on health promotion programs. Health Educ Q. (1988) 15:351–77. doi: 10.1177/109019818801500401 3068205

[56] Xu C Yu J Yang L Li Y Ma D. Intrinsic capacity and health-promoting lifestyle in older adults: a latent class analysis. Front Public Health. (2025) 13:1634373 16 Jul. doi: 10.3389/fpubh.2025.1634373, 40740367 PMC12307196

[57] Teraoka S Hayashida N Shinkawa T Taira Y Nagai-Sekitani Y Irie S . Good stress management capability is associated with lower body mass index and restful sleep in the elderly. Tohoku J Exp Med. (2013) 229:5–10. doi: 10.1620/tjem.229.5, 23196278

[58] Zhu L Shen X Shi X Ouyang X. Factors associated with intrinsic capacity impairment in hospitalized older adults: a latent class analysis. BMC Geriatr. (2024) 24:494. doi: 10.1186/s12877-024-05093-z, 38840051 PMC11151595

[59] Wang K Marbut AR Suntai Z Zheng D Chen X. Patterns in older adults' perceived chronic stressor types and cognitive functioning trajectories: are perceived chronic stressors always bad? Soc Sci Med. (2022) 311:115297. doi: 10.1016/j.socscimed.2022.115297, 36063593

[60] Park CL Finkelstein-Fox L Russell BS Fendrich M Hutchison M Becker J. Psychological resilience early in the COVID-19 pandemic: stressors, resources, and coping strategies in a national sample of Americans. Am Psychol. (2021) 76:715–28. doi: 10.1037/amp0000813, 34081505 PMC8595499

[61] Liu M Zhang M Zhou J Song N Zhang L. Research on the healthy life expectancy of older adult individuals in China based on intrinsic capacity health standards and social stratification analysis. Front Public Health. (2024) 11:1303467. doi: 10.3389/fpubh.2023.1303467, 38356656 PMC10865369

[62] Skou ST Mair FS Fortin M Guthrie B Nunes BP Miranda JJ . Multimorbidity. Nat Rev Dis Primers. (2022) 8:48. doi: 10.1038/s41572-022-00376-4, 35835758 PMC7613517

[63] Lu H Dong XX Li DL Nie X-Y Wang P Pan C-W. Multimorbidity patterns and health-related quality of life among community-dwelling older adults: evidence from a rural town in Suzhou, China. Qual Life Res. (2024) 33:1335–46. doi: 10.1007/s11136-024-03608-0, 38353890

[64] Kaliyaperumal K. Guideline for conducting a knowledge, attitude and practice (KAP) study. AECS Illumination (2004) 4:7–9.

[65] Reid G Vassilev P Irving J Ojakäär T Jacobson L Lawrence EG . The usability and reliability of a smartphone application for monitoring future dementia risk in ageing UK adults. Br J Psychiatry. (2024) 224:245–51. doi: 10.1192/bjp.2024.18, 38356396 PMC11443166

[66] Avazzadeh A Khasawneh OY Faloye ST Asadollahi A Nazari M. Role of smart phones in improving psychological well-being and successful ageing of Iranian old women living with technophobia: a randomized controlled trial. BMC Res Notes. (2025) 18:121. doi: 10.1186/s13104-025-07181-8, 40119442 PMC11929283

[67] Milanti A Chan DNS Parut AA So WKW. Determinants and outcomes of eHealth literacy in healthy adults: a systematic review. PLoS One. (2023) 18:e0291229. doi: 10.1371/journal.pone.0291229, 37792773 PMC10550189

[68] Li S Cui G Yin Y Chen L. Health-promoting behaviors mediate the relationship between eHealth literacy and health-related quality of life among Chinese older adults: a cross-sectional study. Qual Life Res. (2021) 30:2235–43. doi: 10.1007/s11136-021-02797-2, 33661455 PMC8298362

[69] Carstensen LL. Socioemotional selectivity theory: the role of perceived endings in human motivation. Gerontologist. (2021) 61:1188–96. doi: 10.1093/geront/gnab116, 34718558 PMC8599276

[70] Li J Song J Zhu XL. Analysis of status quo and influencing factors for health-promoting lifestyle in the rural populace with high risk of cardiovascular and cerebrovascular diseases. BMC Cardiovasc Disord. (2023) 23:118. doi: 10.1186/s12872-023-03129-7, 36890439 PMC9996853

[71] Mrejen M Nunes L Giacomin K. Socioeconomic inequalities in health and healthcare utilization among the elderly in Brazil: results from the 2019 National Health Survey. Public Health. (2024) 226:165–72. doi: 10.1016/j.puhe.2023.11.015, 38071949

[72] Liu W Zheng R Zhang Y Zhang W. Differences in the influence of daily behavior on health among older adults in urban and rural areas: evidence from China. Front Public Health. (2023) 11:1259204. doi: 10.3389/fpubh.2023.1259204, 37869199 PMC10587611

[73] Nissanholtz-Gannot R Peretz-Dayan H. Equal opportunities in aging: income level moderates the relationship between infrequent participation in formal social activities and loneliness among older adults. J Appl Gerontol. (2023) 42:1982–92. doi: 10.1177/07334648231175429, 37231706 PMC10467004

[74] World Health Organization. Active ageing: A policy framework. Geneva: World Health Organization (2002). 12040973

[75] Wang J Xu J Nie Y Pan P Zhang X Li Y . Effects of social participation and its diversity, frequency, and type on depression in middle-aged and older persons: evidence from China. Front Psych. (2022) 13:825460. doi: 10.3389/fpsyt.2022.825460 PMC9085245 35546944

[76] Hong M De Gagne JC Shin H. Social networks, health promoting-behavior, and health-related quality of life in older Korean adults. Nurs Health Sci. (2018) 20:79–88. doi: 10.1111/nhs.12390, 29178182

[77] Suda T Murayama H Sugawara I. Association between participation in social network service groups and offline social networks. Geriatr Gerontol Int. (2024) 24 Suppl 1:279–84. doi: 10.1111/ggi.14818, 38319046

[78] Watt RG Heilmann A Sabbah W Newton T Chandola T Aida J . Social relationships and health related behaviors among older US adults. BMC Public Health. (2014) 14:533. doi: 10.1186/1471-2458-14-533, 24885507 PMC4046043

[79] Ferrer R Klein WM. Risk perceptions and health behavior. Curr Opin Psychol. (2015) 5:85–9. doi: 10.1016/j.copsyc.2015.03.012, 26258160 PMC4525709

[80] Zhang C Liao WF Ma YM Liang CY. Research on older people\u0027s health information search behavior based on risk perception in social networks-A case study in China during COVID-19. Front. public health (2022) 10:946742. doi:10.3389/fpubh.2022.946742 36033751 PMC9400025

