# Supplementary material for: Exploration of subgroups and predictors of health-promoting lifestyle among older adults in the community: a latent profile analysis
Source: Front Public Health. 2026 Feb 26;14:1583243. doi: 10.3389/fpubh.2026.1583243 (PMC12979150; doi:10.3389/fpubh.2026.1583243)
Supplement: Supplementary file 1 [file Data_Sheet_1.pdf]

Supplementary Table S1 Mean scores for each dimension across the three subgroups of health-promoting lifestyles（M）

	Profile1	Profile2	Profile3
Dimension1	1.39-2.36(1.85)	2.49-3.21(2.78)	3.05-3.63(3.32)
Dimension2	1.44-2.45(1.98)	2.39-3.05(2.67)	2.52-3.15(2.83)
Dimension3	1.69-2.53(2.09)	2.58-3.23(2.91)	3.12-3.62(3.39)
Dimension4	1.07-2.18(1.40)	1.32-2.48(1.78)	2.84-3.58(3.17)
Dimension5	1.30-2.07(1.66)	1.98-2.64(2.30)	3.02-3.51(3.27)
Dimension6	1.56-3.80(2.77)	2.25-3.80(3.09)	3.36-3.92(3.69)
HPL	1.07-3.80(1.86)	1.32-3.80(2.48)	2.52-3.92(3.23)

Note. Dimension 1: Interpersonal Support (IS) Item 1-Item 6 (6 items);
Dimension 2: Stress Management (SM) Item 7-Item 13 (7 items);
Dimension 3: Self-Actualization (SA) Item 14- Item 18 (5 items);
Dimension 4: Health Responsibility (HR), Item 19-Item 28 (10 items);
Dimension 5: Physical Activity (PA), Item 29 - Item 32 (4 items);
Dimension 6: Nutrition (NT), Item 33- Item 36 (4 items).

Supplementary Table S2 Collinearity diagnosis of variables

Variables	Standardized Beta Coefficient	T	Significance	Collinearity Statistics	
				Toleranee	VIF
Age	-0.021	-0.561	0.575	0.696	1.436
Gender	-0.042	-1.293	0.197	0.923	1.083
Number of chronic diseases	0.007	0.165	0.869	0.497	2.012
Long-term medication	0.038	0.845	0.399	0.480	2.082
Smartphone usage	-0.273	-7.008	＜0.001	0.649	1.541
Marital status	0.039	1.105	0.270	0.810	1.234
Residential status	-0.112	-3.425	＜0.001	0.929	1.077
Monthly income	-0.132	-3.292	0.001	0.615	1.627
Geriatric activities	-0.203	-5.911	＜0.001	0.837	1.194
Household registration	-0.089	-2.434	0.015	0.740	1.351
Medical insurance	-0.018	-0.566	0.572	0.934	1.071
Friend network	0.260	6.868	＜0.001	0.689	1.451
Health risk perception	0.038	1.144	0.253	0.887	1.128
